# tRNA Gene Identity Affects Nuclear Positioning

**DOI:** 10.1371/journal.pone.0029267

**Published:** 2011-12-19

**Authors:** Chris D. M. Rodley, Dave A. Pai, Tyrone A. Mills, David R. Engelke, Justin M. O'Sullivan

**Affiliations:** 1 Institute of Natural Sciences, Massey University, Auckland, New Zealand; 2 Department of Biological Chemistry, The University of Michigan, Ann Arbor, Michigan, United States of America; Texas A&M University, United States of America

## Abstract

The three-dimensional organization of genomes is dynamic and plays a critical role in the regulation of cellular development and phenotypes. Here we use proximity-based ligation methods (*i.e.* chromosome conformation capture [3C] and circularized chromosome confrmation capture [4C]) to explore the spatial organization of tRNA genes and their locus-specific interactions with the ribosomal DNA. Directed replacement of one lysine and two leucine tRNA loci shows that tRNA spatial organization depends on both tRNA coding sequence identity and the surrounding chromosomal loci. These observations support a model whereby the three-dimensional, spatial organization of tRNA loci within the nucleus utilizes tRNA gene-specific signals to affect local interactions, though broader organization of chromosomal regions are determined by factors outside the tRNA genes themselves.

## Introduction

Structural genome organization is manifested on different levels, such as linear arrays of genes and spatial arrangement of chromosome territories [1]. Recent studies have implicated interactions that form between genomic loci in the regulation of genes [2]–[4] and of cellular processes such as development [5].

Examination of the spatial organization of gene families can provide insight into how position relates to evolutionary or functional imperatives. The largest family of co-regulated genes in the eukaryotic genome is the RNA polymerase III (Pol III)-transcribed tRNA gene family. The budding yeast *Saccharomyces cerevisiae* has 274 tRNA genes that are dispersed throughout the linear maps of the 16 chromosomes. Fluorescence *in situ* hybridization (FISH) microscopy has shown that these tRNA genes are clustered throughout the cell cycle, with the assistance of condensin complexes bound at each gene, and that clusters localize to the boundary of the nucleolus in a microtubule-dependent manner [6]–[8]. Condensin has also been localized to the nucleolar ribosomal DNA (rDNA) repeats, and mutants of condensin affect proper compaction of the rDNA repeats [8]–[12]. Clustering of tRNA genes has also been observed in fission yeast [13], [14], although their subnuclear localization is different from that seen in *S. cerevisiae*. Proximity-based ligation methodologies, which cross-link spatially adjacent loci, now permit investigation of direct physical interactions among genes in greater detail. Two of these techniques, Genome Conformation Capture (GCC) and a variant of HiC, have previously been used to produce a yeast genome contact map [15], [16] and confirm microscopy results by showing preferential interactions between tRNA genes [15], consistent with the physical clustering observed using fluorescent microscopy.

Since the localization of a large number of dispersed genes to a single subnuclear region necessarily requires a vast rearrangement of the genome, it is of interest to investigate whether individual tRNA gene associations are a controlling influence on the overall organization of the genome, or merely serve as non-specific “fasteners,” providing some level of local condensation, while global organization is determined by other factors. Here we use three methods that rely on proximity—GCC, chromosome conformation capture (3C), and circularized chromosome conformation capture (4C)—to examine the contributions that tRNA genes make to the positioning of specific loci within the *S. cerevisiae* nucleus.

## Results and Discussion

### Genomic fragments that contain tRNA genes are spatially associated with the nucleolus

**Figure 1:**
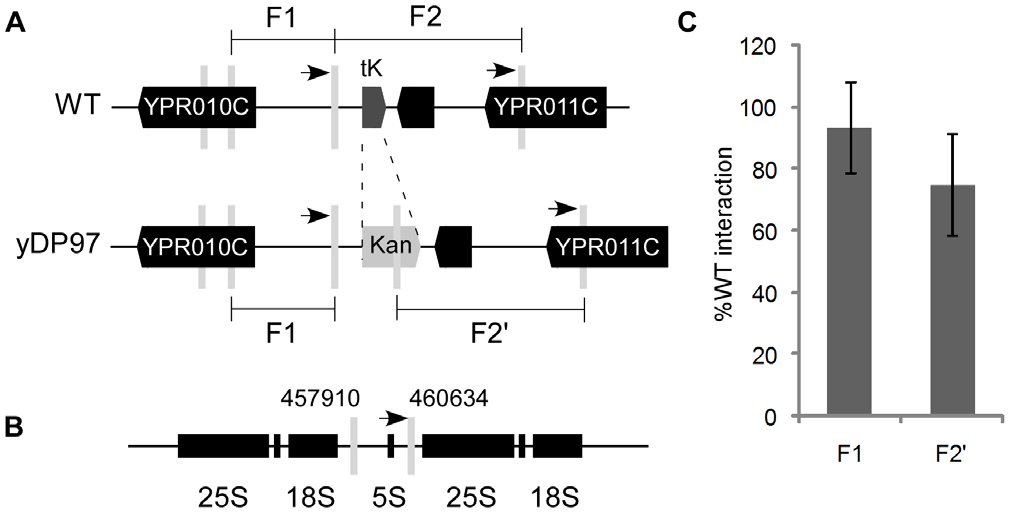
Deleting tRNA^Lys^ *tK(CUU)P* from Chromosome XVI does not significantly perturb strong interactions between this locus and the ribosomal DNA repeats on Chromosome XII. A) Wild type (WT) *S. cerevisiae* strain (BY4741) had the lysine tRNA *tK(CUU)P* gene replaced with the Kan MX6 expression cassette. B) The 3C partner sequence was located across the 5S rDNA (Supplemental information). Grey bars denote *Hin*dIII restriction sites, arrows denote 3C primer positions. C) Quantitative 3C PCR demonstrates no significant reduction in interaction frequency for the F1 or F2/F2' fragments due to removal of the tRNA gene. Results are expressed as percentage of WT F1 or F2 interactions and *GAL1* was used to standardise between samples [15].

GCC using unsynchronized *S. cerevisiae* revealed that many tRNA genes formed multiple interactions with the ribosomal DNA locus (RDN) on chromosome XII [15], which contains multiple tandem copies of the ribosomal RNA genes and forms the nucleolus. While numerous of these interactions were well above background, there was an extremely frequent interaction between one particular DNA fragment containing a lysine tRNA gene on chromosome XVI, *tK(CUU)P* (Chr XVI: 581,025-583,522, Figure 1A), and the non-transcribed spacer sequence (NTS1) in the RDN locus, adjacent to the Pol III-transcribed 5S rRNA gene (Chr XII: 460,025-460,609). None of the *Msp*I restriction fragments adjacent to the *tK(CUU)P* lysine tRNA gene fragment interacted with NTS1. In fact only two of the nearby fragments (Chr XVI: 585884-589137 and 549477-580469, respectively) interacted with the rDNA at levels even slightly above the background (Figure S1, Table S1, and Supplementary statistics in Methods S1). It is theoretically possible that some of the *tK(CUU)P*-NTS1 interactions involve the extra-chromosomal rDNA circles that are present within the yeast nucleus [17], [18]. However, preliminary data indicate that high ERC copy number does not correlate with increased interaction frequencies (data not shown). We conclude that the interaction is driven by signals within the fragment and, given that tRNA genes are known to cluster with the nucleolus [6]–[8], we hypothesized that the tRNA gene was responsible for this interaction.

### Does the lysine tRNA sequence direct the interaction with NTS1?

Clustering of the tRNA genes is mediated by one or more condensin complexes bound to the tRNA genes [8], [19]. Since condensin is also bound to the rDNA repeats and concentrated adjacent to the 5S rRNA gene [19], we hypothesized that the *tK(CUU)P*-NTS1 association might be determined by a condensin-condensin interaction between the tRNA gene complex and the NTS1 region. However, we remained open to the possibility that there might be other contributors that direct this particular tRNA gene to this specific region of the RDN locus. Therefore, to examine to what extent the tRNA gene was responsible for this tight association, we performed 3C on both a wild type (WT) strain and the same strain from which only the tRNA gene coding region, including its intragenic transcription promoter, had been precisely deleted (yDP97, *tK(CUU)P::kanMX6* [20]; Figure 1). Quantitative analysis of locus proximities by 3C [15], [21] showed that precise deletion of the *tK(CUU)P* tRNA coding sequence did not significantly alter the frequency of interaction between the general locus (fragments F1 or F2' on Chromosome XVI) and a *Hin*dIII fragment spanning the 5S rRNA gene and including the NTS1 region (Figure 1B). Furthermore, the deletion did not significantly affect growth rate, determined by co-culturing the two strains for 100 generations (not shown). This lack of growth defect suggests an absence of serious disruption to nuclear organization, in contrast to the strong growth defects that were previously observed in mutants that disrupted general tRNA gene clustering or nucleolar organization [6], [8], [22]. Together, these results indicate that the *tK(CUU)P* lysine tRNA gene itself does not provide the major driving force that determines the proximity of this chromosomal segment to the rDNA repeats. Rather, the general spatial arrangement of these chromosomal loci is driven by factors outside the tRNA gene. Despite this, this result does not preclude the possibility that the tRNA gene might determine the positions of local contacts.

### There are internal and external drivers for tRNA spatial positioning

**Figure 2:**
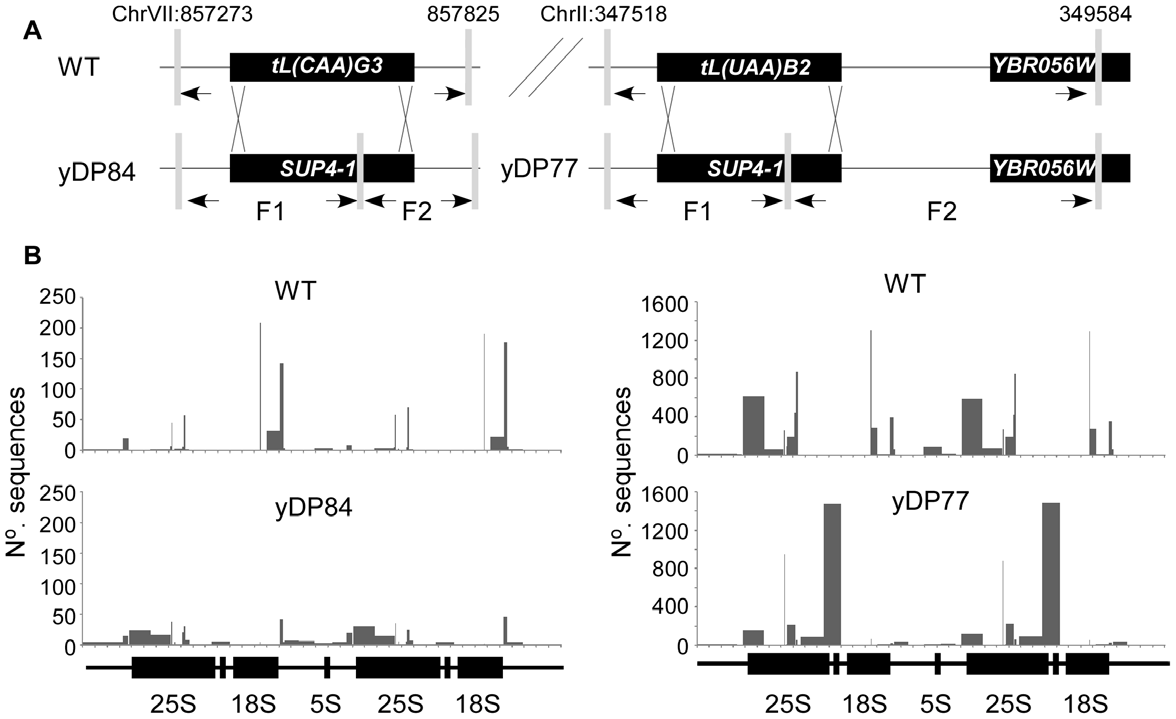
tRNA identity dictates interaction patterns with the ribosomal DNA locus. A) Two leucine tRNA genes (tL(CCA)G3 and tL(UAA)B2), which are located on separate chromosomes, were replaced with the tyrosine tRNA *SUP4*-1 suppressor gene to generate strains yDP84 and yDP77. Grey bars denote *Msp*I restriction sites, arrows denote 4C primer positions. B) Interactions with the rDNA locus were identified by 4C coupled to high through-put sequencing (Methods S1). Raw interaction frequencies have been plotted across a tandem rDNA repeat for clarity. Note, only patterns of interaction along the rDNA locus should be compared as inter-experiment variation has not been corrected for.

The coding regions of yeast tRNA genes contain the major transcriptional promoter elements and are bound by identical sets of transcription components (*i.e.* TFIIIC, TFIIIB, and Pol III) [23]–[25], consistent with the finding that all tested tRNA gene loci can be expressed. Therefore, it was predicted that the nature of the tRNA coding sequences would not alter the interaction behaviour of a locus. To test this prediction, we precisely replaced the mature tRNA coding regions of two different leucine tRNA loci—*tL(UAA)B2* on Chromosome II, and *tL(CAA)G3* on Chromosome VII. The coding regions of these leucine tRNA genes interacted with the RDN locus in the parental strain, although with different patterns across the rDNA repeats (Figure 2B). The *tL(UAA)B2* and *tL(CAA)G3* tRNA coding regions were precisely replaced with the coding region from a tyrosine tRNA gene-variant, the *SUP4*-1 ochre suppressor [26], to allow selection for the insertion (Figure 2A). In each case the 5′ and 3′ flanking regions, including upstream transcription initiation and downstream termination sites, and the primary transcript processed leader and trailer sequences were retained from the original leucine tRNA locus. As expected, the *SUP4*-1 tyrosine tRNA gene replacements of the leucine tRNA coding regions continued to allow association of both loci with the RDN locus, yet unexpectedly the pattern of preferred positions of the associations along the RDN locus were altered. As a control for general disruption of tRNA gene contacts, quantitative 3C analyses of the *S. cerevisiae* yPH499, yDP77, and yDP84 strains identified no significant differences in the interactions between the wild-type *SUP4* tyrosine tRNA gene (*tY(GUA)J2*) (Chr X: 542960-543119) and the 25S rDNA (Chr XII: 451928-452600) locus (Figure S2). These results are consistent with the *tK(CUU)P* lysine tRNA gene replacement data, above, suggesting that external sequences specify general positioning within the nuclear space but that internal factors affect precise local positioning.

By contrast, other interactions identified within the dataset demonstrate that the identity of the internal tRNA gene sequences does influence partner selection. For example, some preferred partners of the *SUP4*-1 sequence were gained at the new locus following replacement of the original leucine tRNA gene sequence (Table S2). The most pronounced of these involved interactions between *SUP4-*1 at the new loci and *MED6* or *MED20* (Table S2). This ability of the internal tRNA gene sequences to contribute to binding partner selection is intriguing, though puzzling. However, the overall effect can be explained by the hypothesis that the flanking sequences act to direct a locus to a particular region of the nucleus. Once within this region, partner selection is influenced by the tRNA gene sequence itself.

### How does internal sequence affect tRNA positioning and partner selection?

The effect of the tRNA gene internal sequences on the positioning of the tRNA gene was completely unexpected, given that all tRNA gene transcription complexes bind the same required components to the internal promoters, as far as is known [23]–[25]. However, this phenomenon could have several causes. Firstly, TFIIIC, the transcription factor that initially recognizes the gene, has a bipartite binding site within the tRNA coding region [27]. The spacing between these sites in the original leucine tRNA genes and in the replacement tyrosine tRNA gene (*SUP4*) is slightly different, and the resulting change in the topology or strength of the TFIIIC-DNA interaction might subtly alter its interaction with other binding partners such as condensin. Moreover, since the degree of occupation of all the genomic tRNA genes by TFIIIC, TFIIIB, and Pol III is variable across the genome [24], [25], [28], [29], the nature of the complexes could be changed by having different geometries or stability of occupation by one or more components. A more speculative yet possible explanation might also be that tRNA gene complexes have tRNA-specific protein components due to differences in their transcript sequences. For example, although both leucine tRNAs and tyrosine tRNAs are cleaved at their 5′ and 3′ mature ends early in biosynthesis, the exact order and location of nucleotide modification events is not clear. Because processing reactions are tRNA specific [30], it therefore remains possible that some enzymes associate with tRNA gene complexes through the nascent RNAs in a sequence-dependent manner.

### Conclusion

These findings support a model whereby the locus-specific tRNA transcription complexes serve as “fasteners” to determine local interactions and promote clustering, but that this occurs in combination with other determinants that dominate global nuclear positioning.

## Materials and Methods

### Strain construction

The coding sequence of *tK(CUU)P* in *S. cerevisiae* BY4741 (*MAT*a *his3*Δ*1 leu2*Δ*0 met15*Δ*0 ura3*Δ*0*), including mature coding sequence and intron, was precisely replaced with the *kanMX6* expression cassette by recombination using PCR fragments generated from plasmid pFA6a-*kanMX6* [20] to create yDP97. Transformants were selected on medium containing G418, and exact gene replacement was verified by PCR and sequencing.

The coding sequence and intron of *tL(UAA)B2* (Chr II, coordinates 347583 to 347699) in the wild-type strain (yPH499; *MAT*a *ura3-52 lys2-801_amber ade2-101_ochre trp1-*Δ*63 his3-*Δ*200 leu2-*Δ*1*) was precisely replaced by recombination with a *SUP4*-1 ochre suppressor tyrosine tRNA coding sequence and intron [26] and selection for suppression of the ochre *ade2* mutation to create yDP77. Correct replacement was subsequently confirmed by PCR analysis and sequencing. Similarly, *tL(CAA)G3* (Chr VII, coordinates 857511 to 857374) was precisely replaced in yPH499 using the same method to create yDP84.

### Genome Conformation Capture [15]

Briefly, chromatin was prepared from 15 sets of 10^8^ (*i.e*. a total of 1.36×10^9^) cross-linked, glucose grown *S. cerevisiae* BY4741 cells. Chromatin was digested with *Msp*I (Fermentas) and ligated (T4 ligase; Invitrogen). Crosslinks were reversed in the presence of proteinase K (final concentration 7–11 µg, Roche). Samples were treated with RNase A (final concentration 10 µgml^−1^) prior to purification by phenol:chloroform (1∶1 v/v, three times) and column extraction (Zymo Clean and Concentrator, Zymo Research). Paired-end sequencing (36 bp) was performed on 5 µg DNA using the Illumina Genome Analyzer platform (Allan Wilson Centre, Massey University, New Zealand & Friedrich Miescher Institute for Biomedical Research, Basel, Switzerland). Sequences were deposited with the Gene Expression Omnibus (GEO) accession number GSE30103.

### Chromosome conformation capture (3C) [31]

*S. cerevisiae* strains (*i.e.* WT and yDP97) were grown (30°C, 160 rpm) to an OD_600_ = 0.6 in synthetic complete media containing amino acid supplements and glucose (2% w/v). Chromatin was prepared according to [15] using *Hin*dIII or *Msp*I restriction enzyme. *Hin*dIII cleaves the *kanMX6* expression cassette and thus results in three restriction fragments in yDP97, as opposed to two fragments in the WT. Interactions between F1 and F2 of the WT strain (F1 and F2′ of the yDP97 strain) on Chr XVI, and the rDNA *Hin*dIII fragment (Chr XII: 457,910–460,634 bp) were measured for three biological replicates.

Quantitative 3C analyses [15], [21] were performed by comparison to dedicated standards using FAM labelled BHQ Probes (BioSearch Technologies; Table S3) and Taqman® Gene Expression Master Mix (Applied Biosystems) on an ABI Prism 7000 Sequence Detection System (SDS7000). Samples (2 µl) were analyzed in triplicate in 20 µl reactions (final volume) using primers listed in Table S3. Real-time analyses were performed using a 3-stage program (50°C, 2∶00 min; 95°C, 10∶00 min; 45× [95°C, 0∶15 sec; 60°C, 1∶00 min]). To standardise between samples, *GAL1* copy number was determined by qPCR (Table S3) using Sybr-green and a five stage program (50°C, 2∶00 min; 95°C, 2∶00 min; 40× [95°C, 0∶15 sec; 59.5°C, 0∶30 sec; 72°C, 0∶30 sec]; 55°C, 1∶00; followed by a dissociation analysis).

### Circular Chromosome Confirmation Capture (4C) [21]

*S. cerevisiae* strains (*i.e.* WT, yDP77, and yDP84) were grown (30°C, 160 rpm) to an OD_600_ = 0.6 in synthetic complete media containing amino acid supplements and glucose (2% w/v). Chromatin was harvested and prepared as for 3C samples using the *Msp*I restriction enzyme.

Nested inverse PCR primers (Table S2) were designed to amplify out of the ‘bait’ *Msp*I fragments that contained the intact *tL(UAA)B2* or *tL(CAA)G3* gene. *SUP4*-1 contains an additional *Msp*I site, which was compensated for by performing nested amplifications of the two fragments independently upon the same 4C library. PCR conditions were as follows 1) first round (95°C, 2∶00 min; 35× [95°C, 0∶30 sec; 59°C, 0∶30 sec; 72°C, 2∶00 min]; 72°C, 5∶00) and 2) nested second round (95°C, 2∶00 min; 35× [95°C, 0∶30 sec; 62°C, 0∶30 sec; 72°C, 2∶00 min]; 72°C, 5∶00). The primer annealing temperatures for fragment 1 and 2 of the yDP77 strain were 68.1°C and 60°C, respectively. Nested primers contained unique 6 bp tags (TCTCTG [yPH499 wild type arrangement of the yDP84 strain], TGATGC [yDP84 fragment 1], and AGCACG [yDP84 fragment 2], AGAGAC [yPH499 wild type arrangement of the yDP77 strain], ACAGAG [yDP77 fragment 1], TAGATC [yDP77 fragment 2]) to enable pooling of the 4C PCR products for sequencing (100 bp paired end) on an Illumina Genome Analyser (Allan Wilson Centre, Massey University). Sequences were mapped onto the *S. cerevisiae* S288c genome sequence using Topography v1.19 [15]. Sequence files are available from GEO (series record GSE30103).

Sorting involved some pre-processing of sequence tags. Each of the individual samples was isolated from the sequence files according to its 6 bp tag and primer sequence and trimmed to 34 bp (with the *Msp*I recognition sequence in the centre). The sequences for fragment 1 and 2 for each mutant (yDP77 and yDP84) were pooled. Since *SUP4*-1 shares considerable identity with other tyrosine tRNAs, particularly around the *Msp*I restriction site, the primer sequences which hybridised adjacent to the novel restriction site within this locus could not be uniquely positioned to the bait fragment on the reference genome. Therefore, the unique primer sequences from the opposite ends of the bait fragments were substituted for the 17 bp repetitive sequences that abut the novel restriction site within *SUP4*-1, prior to analysis. Thus sequences that crossed the *SUP4*-1 restriction site within the bait fragments were accurately mapped to either *tL(UAA)B2* or *tL(CAA)G3*, depending on the interaction under investigation. For analysis, adjacent interaction frequencies were used to correct for between sample comparisons. Manipulated datasets are provided as Data S1, S2, S3.

## Supporting Information

Figure S1 **The high frequency interactions between Chr XVI: 581,025-583,522 (fragment 13476) and the NTS1 sequence adjacent to the 5S rDNA are isolated and not mirrored at adjacent sites within Chr XVI.** Critically, of the six fragments which immediately flank fragment 13476, only the -2 and +2 fragments interact with the rDNA. However, neither interact with the same NTS1 fragment (Chr XII:460,025-460,609) and the maximum number of interactions we observed was 3 orders of magnitude lower than for fragment 13476. Global chromosome capture was performed on unsynchronized exponentially growing *S. cerevisiae* cells (Materials and Methods, Methods S1). Interactions that occurred above the experimental false detection rate (Methods S1) were counted and mapped between restriction fragments surrounding fragment 13476 on Chr XVI (illustrated to the left), and the restriction fragments present across the rDNA (illustrated below the graphs for reference). A, map of interaction frequencies between fragment +2 (Chr XVI:585884-589137) and the rDNA locus; B, map of interaction frequencies between fragment 13476 and the rDNA locus; and C, map of interaction frequencies between fragment −2 (Chr XVI:549477-580469) and the rDNA locus.
(DOCX)

Figure S2 **Genetic background does not affect the interaction frequency between the tyrosine tRNA tY(GUA)J2 (Chr X: 543044-542956) locus and the 25S rDNA (Chr XII: 451928-452600).** 3C was performed using *Msp*I on cross-linked chromatin isolated from yPH499, yDP77 and yDP84 cells grown in SC-glucose to an OD_600_ = 0.6±0.14. Interaction frequencies were determined by quantitative 3C analyses using a fluorescent probe and primers that are specific for the tY(GUA)J2 – 25S rDNA interaction (Table S3) and have been corrected for nuclear genome copy number to facilitate inter-strain comparisons (see Methods). Interaction values are expressed as percentages of the yPH499 sample (set at 100%) +/− standard error of the mean (n = 3).
(DOCX)

Table S1 **Interactions which occur above the experimental noise threshold for MspI fragment number 13476 (Chr XVI 581025-583522 bp), and the adjacent three MspI fragments ( 13473 (−3), 13474 (−2), 13475 (−1), 13476, 13477 (+1), 13478 (+2), 13479 (+3)).** These interactions were identified from interaction networks produced by analysis of Genome Conformation Capture (GCC) libraries constructed using the MspI restriction enzyme and chromatin from glucose grown S. cerevisiae cells (Methods S1). Only data which occurs above the experimental noise threshold has been included (see Supplementary Statistics in Methods S1). These tables do not contain adjacent interactions as they are not included in the statistical calculations.
(XLSX)

Table S2 **SUP4-1 directed interactions detected by circularized chromosome conformation capture (4C).** Captured "prey" fragments whose coordinates are outlined in columns F, G and H are listed alongside the 4C bait fragments (which contain either the wildtype tL(UAA)B2, tL(CAA)G3 loci, or these loci replaced with a SUP4-1 sequence) with which they interact. Only interactions which are conserved by the presence of the SUP4-1 sequence are present in this file. These interactions are conserved regardless of the genomic position of the SUP4-1 sequence.
(XLSX)

Table S3 **Primer and probe sequences used in this study.**
(DOCX)

Data S1 **Comparison of interactions for wild-type and yDP77 strains.** Topography v1.19 program output for the high throughput sequencing of the 4C PCR product. The complete data set is presented along with tabs in which interactions have been classified according to the feature present on the partner sequence (*i.e.* tRNA, autonomous replicating sequences [ARS], YSCPLASM [2 micron plasmid], ribosomal DNA, Telomeres). The read-me tab explains file specific variations and presents a diagram of the fragments that were analysed to determine the interaction patterns.
(XLSX)

Data S2 **Comparison of interactions for wild-type and yDP84 strains.** Topography v1.19 program output for the high throughput sequencing of the 4C PCR product. The complete data set is presented along with tabs in which interactions have been classified according to the feature present on the partner sequence (*i.e.* tRNA, autonomous replicating sequences [ARS], YSCPLASM [2 micron plasmid], ribosomal DNA, Telomeres). The read-me tab explains file specific variations and presents a diagram of the fragments that were analysed to determine the interaction patterns.
(XLSX)

Data S3 **Comparison of interactions for yDP77 and yDP84 strains.** Topography v1.19 program output for the high throughput sequencing of the 4C PCR product. The complete data set is presented along with tabs in which interactions have been classified according to the feature present on the partner sequence (*i.e.* tRNA, autonomous replicating sequences [ARS], YSCPLASM [2 micron plasmid], ribosomal DNA, Telomeres). The read-me tab explains file specific variations and presents a diagram of the fragments that were analysed to determine the interaction patterns.
(XLSX)

Methods S1 **Supplementary Methods.**
(DOCX)
