# Supplementary material for: tRNA Gene Identity Affects Nuclear Positioning
Source: PLoS One. 2011 Dec 19;6(12):e29267. doi: 10.1371/journal.pone.0029267 (PMC3242769; doi:10.1371/journal.pone.0029267)
Supplement: Figure S1 — The high frequency interactions between Chr XVI: 581,025-583,522 (fragment 13476) and the NTS1 sequence adjacent to the 5S rDNA are isolated and not mirrored at adjacent sites within Chr XVI. Critically, of the six fragments which immediately flank fragment 13476, only the -2 and +2 fragments interact with the rDNA. However, neither interact with the same NTS1 fragment (Chr XII:460,025-460,609) and the maximum number of interactions we observed was 3 orders of magnitude lower than for fragment 13476. Global chromosome capture was performed on unsynchronized exponentially growing S. cerevisiae cells (Materials and Methods, Methods S1). Interactions that occurred above the experimental false detection rate (Methods S1) were counted and mapped between restriction fragments surrounding fragment 13476 on Chr XVI (illustrated to the left), and the restriction fragments present across the rDNA (illustrated below the graphs for reference). A, map of interaction frequencies between fragment +2 (Chr XVI:585884-589137) and the rDNA locus; B, map of interaction frequencies between fragment 13476 and the rDNA locus; and C, map of interaction frequencies between fragment −2 (Chr XVI:549477-580469) and the rDNA locus. (DOCX) [file pone.0029267.s001.docx]

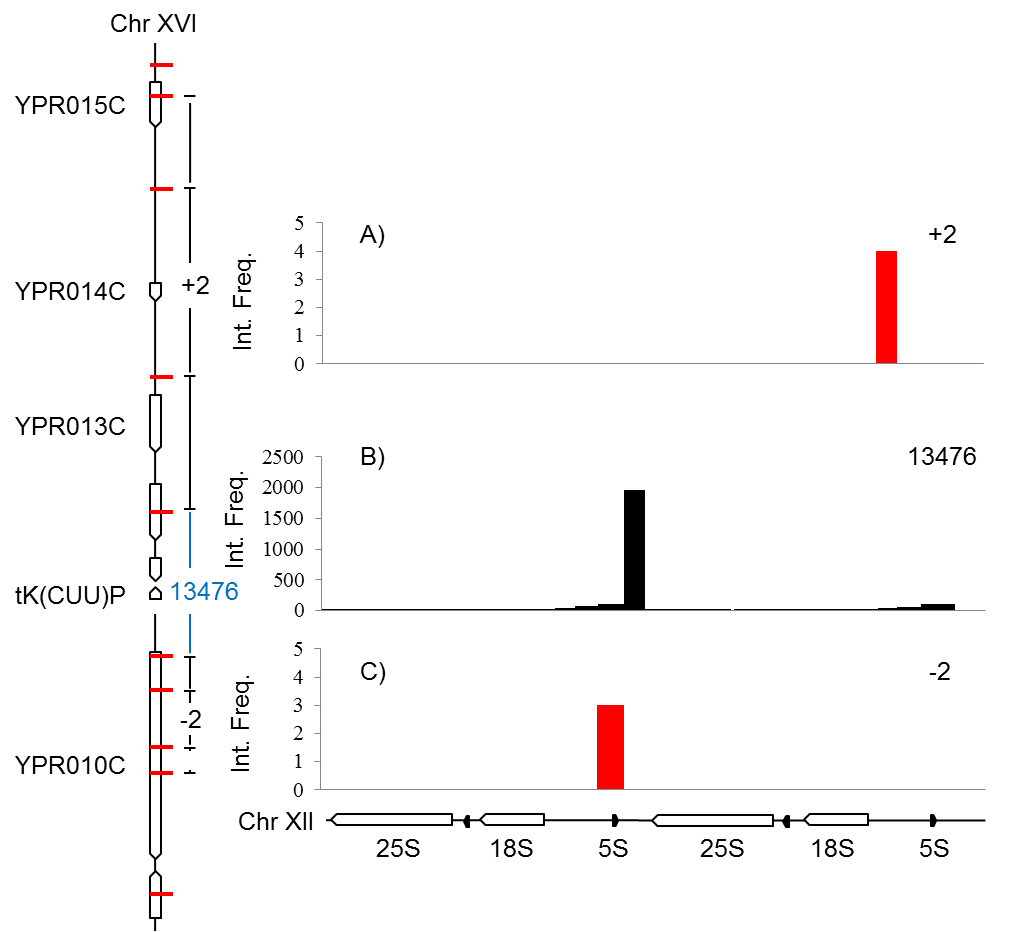


Supplementary Figure 1. The high frequency interactions between Chr XVI: 581,025-583,522 (fragment 13476) and the NTS1 sequence adjacent to the 5S rDNA is isolated and not mirrored at adjacent sites within Chr XVI. Critically, of the six fragments which immediately flank fragment 13476, only the -2 and +2 fragments interact with the rDNA. However, neither interact with the same NTS1 fragment (Chr XII:460,025-460,609) and the maximum number of interactions we observed was 3 orders of magnitude lower than for fragment 13476. Global chromosome capture was performed on unsynchronized exponentially growing *S. cerevisiae* cells (Materials and Methods, Supplementary methods). Interactions that occurred above the experimental false detection rate (Supplementary methods) were counted and mapped between restriction fragments surrounding fragment 13476 on Chr XVI (illustrated to the left), and the restriction fragments present across the rDNA (illustrated below the graphs for reference). A, map of interaction frequencies between fragment +2 (Chr XVI:585884-589137) and the rDNA locus; B, map of interaction frequencies between fragment 13476 and the rDNA locus; and C, map of interaction frequencies between fragment -2 (Chr XVI:549477-580469) and the rDNA locus.
