# Supplementary material for: tRNA Gene Identity Affects Nuclear Positioning
Source: PLoS One. 2011 Dec 19;6(12):e29267. doi: 10.1371/journal.pone.0029267 (PMC3242769; doi:10.1371/journal.pone.0029267)
Supplement: Figure S2 — Genetic background does not affect the interaction frequency between the tyrosine tRNA tY(GUA)J2 (Chr X: 543044-542956) locus and the 25S rDNA (Chr XII: 451928-452600). 3C was performed using MspI on cross-linked chromatin isolated from yPH499, yDP77 and yDP84 cells grown in SC-glucose to an OD600 = 0.6±0.14. Interaction frequencies were determined by quantitative 3C analyses using a fluorescent probe and primers that are specific for the tY(GUA)J2 – 25S rDNA interaction (Table S3) and have been corrected for nuclear genome copy number to facilitate inter-strain comparisons (see Methods). Interaction values are expressed as percentages of the yPH499 sample (set at 100%) +/− standard error of the mean (n = 3). (DOCX) [file pone.0029267.s002.docx]

Supplementary Figure 2. Genetic background does not affect the interaction frequency between the tyrosine tRNA tY(GUA)J2 (Chr X: 543044-542956) locus and the 25S rDNA (Chr XII: 451928-452600). 3C was performed using *Msp*I on cross-linked chromatin isolated from yPH499, yDP77 and yDP84 cells grown in SC-glucose to an OD_600_=0.6 ± 0.14. Interaction frequencies were determined by quantitative 3C analyses using a fluorescent probe and primers that are specific for the tY(GUA)J2 – 25S rDNA interaction (Supplementary Table 3) and have been corrected for nuclear genome copy number to facilitate inter-strain comparisons (see Methods). Interaction values are expressed as percentages of the yPH499 sample (set at 100%) +/- standard error of the mean (n=3).
