# Supplementary material for: tRNA Gene Identity Affects Nuclear Positioning
Source: PLoS One. 2011 Dec 19;6(12):e29267. doi: 10.1371/journal.pone.0029267 (PMC3242769; doi:10.1371/journal.pone.0029267)
Supplement: Table S3 — Primer and probe sequences used in this study. (DOCX) [file pone.0029267.s005.docx]

**Supplementary Table 3:** Primer and probe sequences used in this study.

| Primer Name | Primer Sequence | Tag Sequence |
| --- | --- | --- |
| **4C primers (Outside and Nested)** | | |
| WTyDP84outsideF | CGTTGTAACCGAGAGGATGG | - |
| WTyDP84outsideR | GGCGCCTGATTCAAGAAATA | - |
| WTyDP84NestedF | GTCGATAATGGACTTTGCAC | TCTCTG |
| WTyDP84NestedR | TCAGGCAAGAAAGGGAACCG | TCTCTG |
| WTyDP77outsideF | AAGTTTTCAATTATCCAGCTACTGC | - |
| WTyDP77outsideR | GGCCAACCCTCCGTATAAA | - |
| WTyDP77NestedF | ACGCAATATCATAAAAGCAC | AGAGAC |
| WTyDP77NestedR | TTTCTTTAGTAGCTCCTCCG | AGAGAC |
| F1MUTyDP84outsideF | GTTGGTTTAAGGCGCAAGAC | - |
| F1MUTyDP84outsideR | GCAGGCCCACCTTTATTTCT | - |
| F1MUTyDP84nestedF | GATCGGGCGTTCGACTCGC | TGATGC |
| F1MUTyDP84nestedR | CAGGCAAGAAAGGGAACCG | TGATGC |
| F2MUTyDP84outsideF | GCCGTTAAATCCAATCATCC | - |
| F2MUTyDP84outsideR | GGCGCTCCACCTTTATTGTA | - |
| F2MUTyDP84nestedF | GTCGATAATGGACTTTGCAC | AGCACG |
| F2MUTyDP84nestedR | CGAACAAAAAAATCTCCCG | AGCACG |
| F1MUTyDP77outsideF | AGTTGGTTTAAGGCGCAAGA | - |
| F1MUTyDP77outsideR | TTGCAGCACTCTTTCTTTAGTAGC | - |
| F1MUTyDP77nestedF | AGATCGGGCGTTCGACTCG | ACAGAG |
| F1MUTyDP77nestedR | TTTCTTTAGTAGCTCCTCCG | ACAGAG |
| F2MUTyDP77outsideF | GCGGCACTTCGTAAGTTTTC | - |
| F2MUTyDP77outsideR | CCCCCTAATTCCCTTCTTTT | - |
| F2MUTyDP77nestedF | TGATACGCAATATCATAAAAGCAC | TAGATC |
| F2MUTyDP77nestedR | GTATATTAACAAAAAAATCTCCCG | TAGATC |
| **Positive Control and qPCR Standards** | | |
| RPA135+veFrag1_F | CTGCAGAAGAAACACCATC |  |
| RPA135+veFrag1_R | CCGGATGGAGGTTTGTTAAA |  |
| RPA135+veFrag2_F | CCACCACACTACAACCACCA |  |
| RPA135+veFrag2_R | GAGGAAATGGTTTGAACTGCAT |  |
| rDNA+veHindIII_F | ACTCATGTTTGCCGCTCTG |  |
| rDNA+veHindIII_R | CGATGAGGATGATAGTGTGTAAGA |  |
| **qPCR Primers and Probes** | | |
| RT-RPA135Frag1_R | CCACTCAATTCTGAAGACGAAA |  |
| rDNAHindIIIF | GCTCCATGAAGCAAACTGTCC |  |
| RPA135HindIIIFrag2RorF | TTCTTGTCCATTATTGCCATTT |  |
| rDNAHindIIIProbe | CAAATCCTTTCACGCTCGGGAAGC |  |
| rDNA(25S)Fwd | GGACATCTGCGTTATCGTTTAACAG |  |
| rDNA(25S)Probe | ATGTGCCGCCCCAGCCAAACT |  |
| SUP4 Frag_Rev | CTTGAGATCGGGCGTTCG |  |
| **GCC External Ligation Control** | | |
| E.coli211bp3’*MspI*F | GCCAGAAATTCGTCGGTAAG |  |
| E.coli211bp3’*MspI*R | AACCGGTCATTGAAGTATTGA |  |
| Lambda185bp3‟*MspI*F | TTTACAGCGTGATGGAGCAG |  |
| Lambda185bp3‟*MspI*R | ACCAATCCAGCCGGTCAG |  |

Primers were designed using Primer3 [[11](#_ENREF_11)]. qPCR primers and probes were designed on the RealTimeDesign software from BioSearch Technologies.
