# Supplementary material for: tRNA Gene Identity Affects Nuclear Positioning
Source: PLoS One. 2011 Dec 19;6(12):e29267. doi: 10.1371/journal.pone.0029267 (PMC3242769; doi:10.1371/journal.pone.0029267)
Supplement: Methods S1 — Supplementary Methods. (DOCX) [file pone.0029267.s009.docx]

**tRNA gene identity acts as a determinant of gene positioning**

C.D.M. Rodley^1^, D. A. Pai^2^, D. R. Engelke^2,3^, J.M. O’Sullivan^1,4^

^1^ Institute of Natural Sciences, Massey University, New Zealand; ^2^ Department of Biological Chemistry, The University of Michigan, Ann Arbor, MI 48109-0600, USA^. 3^ Graduate Program in Cellular and Molecular Biology, The University of Michigan, Ann Arbor, Michigan 48109, USA, ^4^ Corresponding author. Email: [j.m.osullivan@massey.ac.nz](mailto:j.m.osullivan@massey.ac.nz)

**Methods**

**Sequence tag description (4C’s)**

A total of 73,010,074 100 bp sequences were generated for the pooled 4C libraries, sorted, trimmed (17 bp either side of the *Msp*I restriction site) and mapped onto the *S. cerevisiae* S288C genome using Topography (v1.19; [[1](#_ENREF_1)]). No mismatches were allowed.

Similar numbers of inter- and non-adjacent intra-chromosomal interactions were observed for the *tL(UAA)B2* and *tL(CAA)G3* loci in the wild-type background (233,456 and 250,195, respectively). The tRNA replacement on Chr VII (yDP84) resulted in the most interactions associated with it overall (1,513,787), while the yDP77 mutant resulted in considerably less (173,546) map able interactions.

**Genome Conformation Capture (GCC)**

- **Strains and growth conditions**

*Saccharomyces cerevisiae* strain BY4741 was stored (-80^o^C) and cultured (30^o^C, 160rpm) on synthetic complete (SC) medium containing amino acid supplements and glucose (2% w/v) [[2](#_ENREF_2)]. For Genome Conformation Capture (GCC) *S. cerevisiae* was recovered from -80^o^C on SC glucose (2% w/v) agar (2%) plates for 48 hours prior to starter culture inoculation. Starter cultures were grown (30*°*C, 160rpm, 16h) in SC glycerol lactate. Test cultures were inoculated, from the starter cultures into SC glucose, grown (30°C, 160rpm) and harvested at an optical density (OD_600_) of 0.6.

GCC was performed according to [[1](#_ENREF_1)]. Briefly, chromatin was prepared from 15 sets of 10^8^ (*i.e*. a total of 1.36x10^9^) cross-linked cells. Briefly, chromatin was digested with *MspI* (Fermentas) and ligated (T4 ligase; Invitrogen). Crosslinks were reversed in the presence of proteinase K (final concentration 7-11µg, Roche). Samples were treated with RNase A (final concentration 10µgml^-1^) prior to purification by phenol:chloroform (1:1 v/v, three times) and column extraction (Zymo Clean and Concentrator, Zymo Research). Paired-end sequencing (36 bp) was performed on 5μg DNA using the Illumina Genome Analyzer platform (Allan Wilson Centre, Massey University, New Zealand & Friedrich Miescher Institute for Biomedical Research, Basel, Switzerland).

External controls were added at two steps in the GCC protocol to control for random ligation events. The first ligation control, a linear DNA fragment with a free *MspI* site at one end, was added in a 1:1 ratio with the nuclear genome prior to the addition of ligase. The second ligation control (1x10^6^ molecules of pUC19) was included prior to RNase A treatment as a control for the sequencing step ligation.

**External controls for random ligation events**

To experimentally control for spurious inter-molecular ligation events, during the GCC process, samples were spiked with two ligation controls during library preparation. The first ligation control consisted of PCR products (Supplementary Table 3) that were added (1:1 ratio with the nuclear genome copy number) before the GCC ligation step. These controls were designed to estimate the frequency of random inter-molecular ligation events during GCC library preparation. A maximum of 47 separate ligation events were observed, none of which occurred at levels above the statistically defined experimental noise. The second ligation control consisted of the addition of pUC19 plasmid to the sample following the GCC ligation in order to control for random ligation events during preparation of the samples for sequencing, at the sequencing centre. We observed a maximum of six interactions between pUC19 and the rest of the genome; again none of these interactions were above the statistically defined experimental noise. In conclusion, the fact that the high copy number rDNA and mitochondrial DNA elements do not show significant levels of random inter-molecular interactions with our internal control sequences is empirical evidence that the interactions we observe result from intra-molecular ligation events. Therefore, random ligation events during sample preparation do not account for the interactions we observe.

External controls were produced by the PCR amplification of short regions from the *E. coli* genome. Primers (Supplementary Table 3) were designed to include an *MspI* site at one end of the final product. PCR products were digested with *MspI* (37°C, 2hrs) prior to column purification (Zymo Clean and Concentrator). Purified, digested PCR products were introduced into the GCC samples at a 1:1 ratio with the nuclear genome (unique region) prior to the ligation step during GCC preparation of the sample. The *E. coli* fragment was introduced into the glucose sample and resulted in 10 ligation events to *MspI* restriction fragments including eight regions on the nuclear genome, one from the YSCPLASM and one from the mitochondrial genome. No ligation events between the *E. coli* external ligation control and the *S. cerevisiae* genome occurred at levels deemed significant.

**Sequence Population Statistics**

We wanted to know whether the sequences obtained from the Illumina Genome Analyser were representative of the *S. cerevisiae* genome. From published reports of copy number [[3-5](#_ENREF_3)] we calculated how many base pairs the whole genome complement is and therefore what percentage specific genomic elements should encompass (Supplementary Methods Table 1). The number of base pairs in the yeast genome complement was calculated by the addition of the 16 yeast chromosomes at a copy number of 1, the rDNA repeats (9.1kb) at a copy number of 200, the mitochondrial genome and 2 micron plasmid at a copy number of 50, for a total of 18,563,326 bp. The sequence files were aligned against the *S. cerevisiae* genome to determine the total number of sequences which could be aligned. We then aligned the sequences against a number of different genomic features to compare the percentage the particular feature should make up against the percentage of sequences which could be aligned against the same feature. The reference genes (housekeeping) were obtained by batch download from the SGD website. The set consisted of 646 verified ORFs across all chromosomes that were between 400-500 amino acids in size. Sequences are available upon request. Supplementary Methods Table 1 summarises the sequence population statistics.

**Supplementary Methods Table 1:**

|  | **% of Genome (18,563,326)^a^** | **% of Glucose Sequences (101,767,324)^†^** |
| --- | --- | --- |
| **CEN** | 0.010 | 0.004 |
| **TEL** | 0.758 | 1.319 |
| **rDNA 200** | 9.804 | 15.943 |
| **Housekeeping** | 4.755 | 6.702 |
| **YSCPLASM (50)** | 1.702 | 2.428 |
| **Mitochondria (50)** | 23.104 | 3.207 |

^a^ The size of the *S. cerevisiae* genome in base pairs including 200 rDNA repeats, 50 mitochondria, and 50 2-micron plasmids.

**^†^** The subset of sequences which could be positioned on the *S. cerevisiae* genome with no mismatches.

**GCC Network Assembly**

Network assembly was performed using Topography v1.19 (available on request [[1](#_ENREF_1)]). The SOAP [[6](#_ENREF_6)] algorithm was used to position paired end sequences and single ends, which contain an *MspI* restriction site, onto the *S. cerevisiae* reference genome. No mismatches were allowed. The two biological repeats were highly correlated for statistically significant interactions (R^2^=0.78). Accordingly, sequences from biological repeats were combined and reanalyzed.

***Saccharomyces cerevisiae* reference genome used in the SOAP Alignment**

The reference genome consists of the 16 *S. cerevisiae* nuclear chromosomes (NC_001133.7, NC_001134.7, NC_001135.4, NC_001136.8, NC_001137.2, NC_001138.4, NC_001139.8, NC_001140.5, NC_001141.1, NC_001142.7, NC_001143.7, NC_001144.4, NC_001145.2, NC_001146.6, NC_001147.5, NC_001148.3) as well as the mitochondrial genome (NC_001224.1) and the 2-micron plasmid (YSCPLASM), pUC19 and the ligation control sequences.

**Supplementary Statistics**

Statistical calculations were performed on datasets in which the sequences could be uniquely positioned on the reference genome. Where the analysis was concerned with connections between elements considered repetitive (*i.e*., the rDNA, mtDNA and 2-micron plasmids) the copy numbers of the elements was corrected for. Connections between unique sequences and connections involving repetitive elements were analyzed separately. Values for copy number correction were determined from: 1) published reports of the frequencies of these elements within *S. cerevisiae* [[3-5](#_ENREF_3)]; and 2) the frequency of these elements in the GCC sequence files that were returned from the sequencing centres.

During the preparation of the GCC samples random ligation events occur. These events occur during the ligation following restriction enzyme digestion and during the sequencing preparation steps (*i.e*., linker addition during sequencing library preparation). Thus, our null hypothesis is that all interactions we identified are a result of random ligation events, or that there are only random ligations. In an attempt to control for this, two steps were included in our analyses. 1) External controls were added during the GCC library preparation to obtain estimates of the rates of inter-molecular ligation events. 2) We perform statistical analyses to determine whether our GCC dataset is something other than random. To achieve this we performed 100,000 simulations of random pairings (we performed these simulations previously [[1](#_ENREF_1)] to establish the GCC method, but have repeated them here for these new datasets). Each individual simulation contained the same total number of interactions as the GCC dataset.

The *in silico* simulations (100,000) were performed [[1](#_ENREF_1)] to determine the maximum count of a particular interaction that would be observed under this noise model, given the same number of sequences, interactions and fragments as in the experimental data. The results of the simulations lead us to conclude that the interaction patterns cannot be attributed to noise alone with a p-value less than 10^-5^.

Secondly, we performed analyses to determine what frequency individual interactions have to achieve before they are deemed to be present at a level above experimental noise. It is justified to assume the pairings are independent and therefore the number of times one specific pairing occurs is a binomially distributed random variable. Let S1 and S2 be the number of mitochondrial and nuclear segments, respectively, which participate in at least one interaction. We calculate the probability P(X≥k) where N is number of observed pairings and p is 1 divided by S1 multiplied by S2, for one specific pairing to occur k or more times. L is then S1 multiplied by S2, being the number of possible pairings and we expect to see L∙P(X≥k) pairings occurring k or more times by chance. This provides us with the expected number of false positives. The value k has to be chosen in such a way to provide an acceptable number of false positives and consequently an acceptable false positive rate (see Supplementary Methods Table 2). k=3 was selected as an acceptable noise cut-off value for the glucose GCC sample (Bold) when the rDNA is under investigation. The copy number of the rDNA fragments which was included in the experimental noise cut off calculations has been determined below (Statistical Analysis of Repetitive elements).

**Supplementary Methods Table 2:**

| **Sample** | **k** | **N** | **p** | **P(X≥k)** | **L** | **L∙P(X≥k) Expected number of false positives** | **False Positive Rate** |
| --- | --- | --- | --- | --- | --- | --- | --- |
| **Glucose** | 3 | 478978 | 1/(13622*6377.41) | 2.78E-08 | 86873079 | 2.4167 | 0.000392516 |

**Statistical Analysis of Repetitive elements**

Repetitive elements are those genomic features which occur more than once within the genome (*e.g.,* the mitochondrial genome, the rDNA and 2 micron plasmid). We were expressly interested in the interactions between the rDNA repeats and the rest of the genome (expressly, segment number 13476). We calculated the copy number of the rDNA repeats in our samples by aligning the sequence files against a short section of rDNA ([rDNA]Chr XII: 460517-460612) (Supplementary Methods Table 3) and comparing it against the single copy GAL1 locus ([Gal]Chr II: 279790-279909). We calculated the ratio of rDNA to the unique nuclear element (GAL1). The calculated copy number ratio is outlined in Supplementary Methods Table 4.

**Supplementary Methods Table 3:**

| **Gal (146bp)** | **Sequence Reads** | **Aligned Reads** | **Percentage** |
| --- | --- | --- | --- |
| Glucose | 112335584 | 733 | 0.00065251 |
| **rDNA (96bp)** | **Reads** | **Aligned Reads** | **Percentage** |
| Glucose | 112335584 | 103881 | 0.09247381 |

**Supplementary Methods Table 4:**

|  | **Gal:rDNA** |
| --- | --- |
| **Glucose** | 141.7203274 |

**References**

1. Rodley CD, Bertels F, Jones B, O'Sullivan JM (2009) Global identification of yeast chromosome interactions using Genome conformation capture. Fungal Genet Biol 46: 879-886.

2. Kaiser CSMMA (1994) Methods in Yeast Genetics, A Cold Spring Harbor Laboratory Course Manual. Cold Spring Harbor, NY.: Cold Spring Harbor laboratory press.

3. Cui H, Ghosh SK, Jayaram M (2009) The selfish yeast plasmid uses the nuclear motor Kip1p but not Cin8p for its localization and equal segregation. The Journal of Cell Biology 185: 251-264.

4. Williamson DH, Fennell DJ, editors (1979 ) Methods in Enzymology: Academic Press. 728-733. p.

5. Kobayashi T, Heck DJ, Nomura M, Horiuchi T (1998) Expansion and contraction of ribosomal DNA repeats in Saccharomyces cerevisiae: requirement of replication fork blocking (Fob1) protein and the role of RNA polymerase I. Genes Dev 12: 3821-3830.

6. Li R, Li Y, Kristiansen K, Wang J (2008) SOAP: short oligonucleotide alignment program. Bioinformatics 24: 713-714.
